# Antitrypanosomal Alkaloids from the Marine Bacterium *Bacillus pumilus*

**DOI:** 10.3390/molecules170911146

**Published:** 2012-09-18

**Authors:** Sergio Martínez-Luis, José Félix Gómez, Carmenza Spadafora, Héctor M. Guzmán, Marcelino Gutiérrez

**Affiliations:** 1 Center for Biodiversity and Drug Discovery, Institute for Scientific Research and High Technology Services, City of Knowledge, P.O. Box 0843-01103, Panama; Email: smartinez@indicasat.org.pa; 2 Departamento de Química, Centro de Investigación y de Estudios Avanzados del IPN, México D.F. 07360, Mexico; Email: jfgomez@cinvestav.mx; 3 Center for Cellular and Molecular Biology of Diseases, Institute for Scientific Research and High Technology Services, City of Knowledge, P.O. Box 0843-01103, Panama; Email: cspadafora@indicasat.org.pa; 4 Smithsonian Tropical Research Institute, Balboa, Ancon, P.O. Box 0843-03092, Panama; Email: guzmanh@si.edu

**Keywords:** indole alkaloids, *Trypanosoma cruzi*, *Bacillus pumilus*, Chagas disease

## Abstract

Fractionation of the ethyl acetate extract of the marine bacterium *Bacillus pumilus* isolated from the black coral *Antipathes* sp. led to the isolation of five compounds: *cyclo*-(*L*-Leu-*L*-Pro) (**1**), 3-hydroxyacetylindole (**2**), *N*-acetyl-β-oxotryptamine (**3**), *cyclo-*(*L*-Phe-*L*-Pro) (**4**), and 3-formylindole (**5**). The structures of compounds **1**−**5** were established by spectroscopic analyses, including HRESITOF-MS and NMR (^1^H, ^13^C, HSQC, HMBC and COSY). Compounds **2**, **3** and **5** caused the inhibition on the growth of *Trypanosoma cruzi* (*T. cruzi*), with IC_50_ values of 20.6, 19.4 and 26.9 μM, respectively, with moderate cytotoxicity against Vero cells. Compounds **1**−**5** were found to be inactive when tested against *Plasmodium falciparum* and *Leishmania donovani*, therefore showing selectivity against *T. cruzi* parasites.

## 1. Introduction

Chagas disease, also known as American trypanosomiasis, is a tropical neglected disease that largely affects populations in Latin America, where the disease is endemic. An estimated 10,000 people died in 2008 from the disease and more than 25 million people are at risk of infection [1].

This illness occurs in two phases. The initial or acute phase takes about two months post-infection. In most of the cases symptoms are either absent or mild. Less than 50% of people only display a skin lesion or purple swollen eyelids on an eye. During the second or chronic phase, as many as 30% of patients develop heart disease, and up to 10% of patients develop gastrointestinal disturbances, neurological problems or a combination of both. In subsequent years the infection may result in sudden death or heart failure due to progressive destruction of the heart muscle [2].

Current treatment against Chagas disease is based on toxic chemotherapeutic agents, specifically benznidazole and nifurtimox, which have many serious side effects. Both drugs are almost 100% effective during the acute phase. Nevertheless, their effectiveness decreases significantly during the chronic phase. Moreover, apart from the adverse reactions that usually arise with them (incidence of up to 40% of patients) [2,3], the treatment is long in duration, increasing patients’ non-compliance.

The poor therapeutic options available for the treatment of Chagas disease demand an urgent search and development of new and more effective anti-*Trypanosoma cruzi* (anti-*T. cruzi*) drugs. In this regard, natural products offer a diversity of possibilities for drug discovery against Chagas disease. To date most of the efforts have focused on plant metabolites [4,5,6]. However, the marine environment remains largely unexplored as a resource for antitrypanosomal compounds. A few examples found in a literature search on marine organisms producing anti-*T. cruzi* metabolites include sponges [7,8,9], tunicates [10], fungi [11,12] and marine cyanobacteria [13,14,15]. Remaining marine taxa, including heterotrophic bacteria, remain untapped as a source of compounds against Chagas disease.

Among marine heterotrophic bacteria, the genus *Bacillus* has been explored for its potential to produce active secondary metabolites. Most of the compounds isolated from this genus, including macrolides [16,17,18], diketopiperazines [19], lipoamides [20] and fatty acids [21], showed antimicrobial activity. Moreover other *Bacillus* metabolites belonging to the cyclopeptides [22,23,24], isocoumarins [25] and thiazole alkaloids [26] structural classes showed anticancer, antimalarial and algicidal activity, respectively. No anti-*T*. cruzi compounds have been reported from the genus *Bacillus*.

This work was designed to investigate the potential of heterotrophic bacteria associated with Panamanian corals as a source of antiprotozoal compounds. Herein we report the isolation, identification and anti-parasitic activity of compounds produced by the bacterium *Bacillus pumilus* isolated from the black coral *Antiphates* sp, collected near Otoque Island, Pacific coast of Panama.

## 2. Results and Discussion

### 2.1. Collection and Identification of Biological Material

The strain GL0057 was isolated from the mucus of the black coral *Antiphates* sp. collected near Otoque Island in the Golf of Panama (Pacific Ocean). The bacterium was identified based on its 16S rRNA sequence which showed 99% similarity to *Bacillus pumilus* when compared with sequences deposited in BLAST-NCBI database. The sequence of strain GL0057 was deposited in GenBank under accession number JX569793.

### 2.2. Extraction, Isolation and Structural Elucidation of Compounds

**Figure 1:**
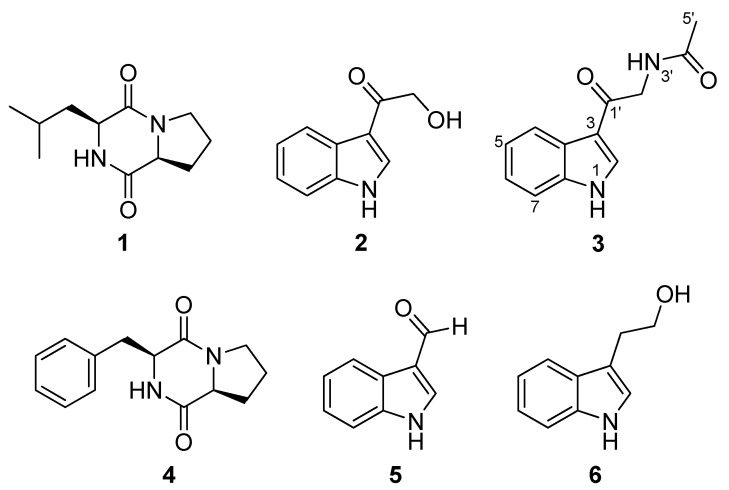
Chemical structuresof metabolitesproduced by *Bacillus pumilus*.

Strain GL0057 was cultured in 10 L liquid media and extracted with ethyl acetate to obtain the organic crude extract. The extract was fractionated using C-18 solid phase extraction cartridges (SPE) followed by HPLC purification obtaining and identifying the following compounds: *cyclo-*(*L*-Leu-*L*-Pro) (**1**) [27], 3-hydroxyacetylindole (**2**) [28], *N*-acetyl-β-oxotryptamine (**3**) [29], *cyclo-*(*L*-Phe-*L*-Pro) (**4**) [27], and 3-formylindole (**5**) [30] (Figure 1). Structures of compounds **1–5** were defined by spectroscopic analysis including HRESITOF-MS, ^1^H-NMR, ^13^C-NMR and optical rotations, and we found them to be metabolites previously reported in the literature. NMR assignments for compounds **2, 3** and **5** were confirmed by 2D-NMR experiments including HSQC, HMBC and COSY.

Compound **3** was isolated as an amorphous white solid. HRESITOF-MS showed a pseudomolecular ion peak [M+Na]^+^ at *m/z* 239.0790, corresponding with the molecular formula C_12_H_12_N_2_O_2_Na (calcd. for C_12_H_12_N_2_O_2_Na, 239.0791). The ^1^H-NMR spectrum showed characteristic chemical shifts of a mono-substituted indole nucleus at δ_H_ 7.25 (2H, m, H-5, H-6), 7.48 (1H, m, H-7), 8.24 (1H, bs, H-4) and 8.26 (1H, m, H-2), in addition to one methylene group attached to a nitrogen at δ 4.62 (2H, s) and one acetate methyl group at δ_H_ 2.11 ppm (3H, s, H-5′). The ^13^C-NMR spectrum showed resonances for 12 carbon atoms. DEPT experiments revealed the presence of two quaternary carbonyls at δ_C_ 192.7 (C-8) and 174.5 ppm (C-10), three sp2 quaternary carbons at δ_C_ 139.1 (C-7a), 127.8 (C-3a), 116.7 (C-3); five sp2 methines at δ_C_ 127.8 (C-2), 125.3 (C-6), 124.2 (C-5), 123.5 (C-4), 113.8 (C-7); and the presence of a methylene and a methyl group at δc 47.9 ppm and 23.3 ppm, respectively. The spin system formed by protons H-4 to H-7 was confirmed by COSY experiments. 

The connection of the oxoethyl moiety to the indole nucleus was confirmed through ^2,3^*J* HMBC correlations observed between H-2′ (δ_H_ 4.62) and carbon C-3. The connection between the acetamide group to the oxoethyl was also confirmed through ^2,3^*J* HMBC correlations between H-2′ and C-4′ (δc 174.5 ppm). The presence of an exchangeable amide proton was evidenced by measuring of the ^1^H-NMR spectrum in deuterated methanol and chloroform. Therefore, compound **3** was identified as *N*-acetyl-β-oxotryptamine by its NMR data and comparison with the published data in the literature [29].

The NMR data of compounds **2** and **5** showed similar spectroscopic features as compound **3**, the main differences being found in the length of the side chain. Thus, compound **3** contained an oxoethyl-acetamide group, while compounds **2** and **5** contained a hydroxyacetyl (δc 195.8, C-1′; 66.1, C-2′, δ_H_ 4.75, H-2′) and formyl (δc 188.3, C-1′; δ_H_ 9.88, s, H-1′) groups, respectively.

### 2.3. Antiparasitic Activity

Compounds **1−5** were tested for activity against parasites causative of three neglected tropical diseases: *Leishmania donovani*, *Trypanosoma cruzi* (*T. cruzi*) and *Plasmodium falciparum*. Indole alkaloids **2**, **3** and **5** selectively inhibited the growth of *T. cruzi*, being inactive against *P. falciparum* and *L. donovani* (Table 1). Diketopiperazines **1** and **4** were inactive against all parasites. Compounds **1**, **3** and **5** showed no significant cytotoxicity when evaluated against African Green Monkey kidney epithelial (Vero) cells displaying IC_50_ values of 149, 66 and 87 µM, respectively.

**Table 1:** Biological activity (IC_50_µM) of **1–5** against tropical parasites.

Compounds	*L. donovani*	*P. falciparum*	*T. cruzi* ^a^
*Cyclo*-(*L*-Leu-*L*-Pro) (**1**)	I	I	I
3-Hydroxyacetylindole (**2**)	I	I	20.6
*N*-acetyl-β-oxotryptamine (**3**)	I	I	19.4
*Cyclo*-(*L*-Phe-*L*-Pro) (**4**)	I	I	I
3-Formylindole (**5**)	I	I	26.9
Nifurtimox			1.6

I, Inactive at 10 µg/mL; ^a^ Values for *T. cruzi* are the average of two experiments with two replicates each.

Given the structural similarities and biological activity among compounds **2**, **3** and **5**, the length and substitution of the side chain attached to carbon C-3 does not appear to produce a significant difference in the biological activity. However, if we compare compound **2** with the structure of tryptophol (**6**), which also showed some antitrypanosomal activity (IC_50_ 30.6 µM) [31], it can be inferred that the presence of an electron-attracting group in the side chain, like the carbonyl at C-1′, enhances the biological activity. This observation could be a starting point for designing new analogs based on the activity of these alkaloids.

## 3. Experimental

### 3.1. General Procedures

Optical rotations were measured with a Jasco P-2000 polarimeter. NMR spectra were acquired on a Jeol Eclipse 400 MHz spectrometer and are referenced to residual solvent ^1^H and ^13^C signals (δ_H_ 7.26, δ_C_ 77.0 for CDCl_3_). Low-resolution ESI-MS were acquired on Jeol LC-mate mass spectrometer, while high-accuracy mass measurements were obtained on an Agilent 6230 mass spectrometer. The purification of the compounds was carried out on Agilent 1100 HPLC system equipped with a quaternary pump, a diode array detector, and a reverse phase silica gel column (Phenomenex Synergi Hydro-RP, 250 mm × 100 mm, 4 μm) at a flow rate of 1.0 mL/min. Pre-fractionation of the extract was carried out using Supelclean^TM^ C-18 solid phase extraction (SPE) tubes. TLC was performed on precoated silica gel 60 F254 plates (Merck). All solvents were HPLC grade and used without further purification.

### 3.2. Biological Material Collection and Identification

A healthy specimen of the black coral *Antipathes* sp. was collected by hand using SCUBA near Otoque Island in the Pacific Ocean off the coast of Panama in August 2009. For the isolation of coral-associated bacteria, a small piece of the coral was rinsed with sterile seawater to remove loosely attached bacteria, and a small portion of the coral mucus was inoculated on Petri dishes with seawater-based nutrient media. Agar plates were taken to the laboratory and observed for bacterial isolation, at room temperature over the period of one month. Strain GL0057 was further isolated from the collection plate and successively re-plated until a pure strain was obtained. Coral was identified as *Antiphates* sp. based on its morphology and SEM-micrographs of the coral sclerites by Dr. Hector Guzman from the Smithsonian Tropical Research Institute. Taxonomy of the strain GL0057 was carried out by sequencing of the 1409 bp of 16S rRNA gene. The gene sequence was submitted to NCBI-BLAST database and showed 99% similarity to 16S rRNA gene sequence of a strain identified as *Bacillus pumilus*. Reference specimens of the coral GLOT-120209-03 and the bacterial strain GL0057 are deposited at INDICASAT’s Center for Biodiversity and Drug Discovery.

### 3.3. Fermentation and Extraction

The bacterium *Bacillus pumilus* was inoculated in ten Erlenmeyer flasks (1 L), containing 500 mL of seawater-based liquid medium (10 g of starch of potatoes, 4 g of yeast extract and 2 g of peptone in 1 L of natural sea water). All reagents for the culture media were purchased from Sigma. Erlenmeyer flasks were placed in an orbital shaker at 172 rpm at room temperature for 10 days. After this period, the culture broth was extracted with ethyl acetate (2 L × 3). The organic extract was washed with distillated water (2 L × 3) in order to remove traces of culture media from the organic extract. The extract was then dried under reduced pressure to obtain 456 mg of solid.

### 3.4. Isolation of Alkaloids

Extract (456 mg) was fractionated using C-18 solid phase extraction (SPE) cartridges eluted with a stepwise gradient of 20%, 40%, 50%, 60%, 70%, 80%, 90%, and 100% of methanol in water to yield eight fractions (F1-F8). Fractions F1 to F8 were analyzed by NMR spectroscopy and fraction F2 (eluted with 40% of methanol in water) showed the presence of secondary metabolites in its ^1^H-NMR spectrum. Therefore, fraction F2 was purified by reverse phase HPLC (Synergi Hydro 250 × 10 mm column, isocratic elution of 85% methanol-15% distillated water, UV detector at 254 nm, flow of 1.0 mL/min) to afford 1.8 mg of *cyclo-*(*L*-Phe-*L*-Pro) (**1**), 0.8 mg of 3-hydroxyacetylindole (**2**), 0.9 mg of *N*-acetyl-β-oxotryptamine (**3**), 1.4 mg of *cyclo-*(*L*-Leu-*L*-Pro) (**4**), and 1.0 mg of 3-formylindole (**5**).

### 3.5. Spectral Data

*3-Hydroxyacetylindole* (**2**). Colorless solid; ^1^H-NMR (CD_3_OD): δ: 8.25 (1H, m, H-4), 8.23 (1H, bs, H-2), 7.48 (1H, m, H-7), 7.25 (2H, m, H-5, H-6), 4.75 (2H, s, CH_2_-2′); ^13^C-NMR (CD_3_OD): δ: 195.8 (C-1′), 138.1 (C-7a), 133.8 (C-2), 126.8 (C-3a), 124.2 (C-6), 123.1 (C-5), 122.5 (C-4), 118.7 (C-3), 112.8 (C-7), 66.1 (C-2′); HRESITOF-MS *m/z* 198.0527 [M+Na]^+^ (C_10_H_9_NO_2_Na requires 198.0525). 

*N-Acetyl-β-oxotryptamine* (**3**). Withe solid; ^1^H-NMR (CD_3_OD): δ: 8.26 (1H, bs, H-2), 8.24 (1H, m, H-4), 7.48 (1H, m, H-7), 7.25 (2H, m, H-5, H-6), 4.62 (2H, s, CH_2_-2′), 2.11 (3H, s, CH_3_-5′); ^13^C-NMR (CD_3_OD): δ: 192.7 (C-1'), 174.5 (C-4′), 139.1 (C-7a), 135.1 (C-2), 127.8 (C-3a), 125.3 (C-6), 124.2 (C-5), 123.5 (C-4), 116.7 (C-3), 113.8 (C-7), 47.9 (C-2′), 23.3 (C-5′); HRESITOF-MS *m/z* 239.0790 [M+Na]^+^ (C_12_H_12_N_2_O_2_ Na requires 239.0791).

*3-Formylindole* (**5**). Colorless solid; ^1^H-NMR (CD_3_OD): δ: 9.88 (1H, d, *J* = 2.9, H-1′), 8.25 (1H, m, H-4), 8.23 (1H, bs, H-2), 7.48 (1H, m, H-7), 7.25 (2H, m, H-5, H-6); ^13^C-NMR (CD_3_OD): δ: 188.3 (C-1′), 138.3 (C-2), 136.4 (C-7a), 123.6 (C-3a), 122.3 (C-6), 122.3 (C-5), 121.0 (C-4), 118.8 (C-3), 111.8 (C-7); HRESITOF-MS *m/z* 146.0601 [M+H]^+^ (C_9_H_8_NO requires 146.0600). 

### 3.6. *In Vitro* Biological Activity

#### 3.6.1. *Plasmodium falciparum* Bioassay

Antiplasmodial activity was evaluated using a fluorometric method based on the detection of parasite DNA with the fluorochrome PicoGreen using a chloroquine-resistant strain (Indochina W2) of *P. falciparum*. The samples were considered inactive if they inhibited less than 75% of the growth of parasites as compared to their untreated controls at 10 µg/mL [32].

#### 3.6.2. *Leishmania donovani* Bioassay

Axenically grown (cell free) amastigotes of *L. donovani* were used to assess parasite growth and survival, in the presence of the compounds. Samples were tested in duplicate at 10 µg/mL. The results were expressed as a percentage of growth inhibition (GI) compared to untreated controls. Samples that showed below 75% GI were considered inactive [33].

#### 3.6.3. *Trypanosoma cruzi* Bioassay

*T. cruzi* bioassays were performed using a colorimetric method, and the inhibition of parasite growth was assessed by the expression of the reporter gene for beta-galactosidase (β-Gal) in the recombinant Tulahuen clone C4 of *T. cruzi*. Assays were performed in duplicate on amastigotes, the intracellular form of the parasite infecting African green monkey kidney (Vero) cells, exposed during five days to different concentrations (10, 2, 0.4, 0.08 and 0.016 μg/mL) of the test compounds at 37 °C under an atmosphere of 5% CO_2_/95% air. The resulting color from the cleavage of chlorophenol red-β-D-galactoside (CPRG) by β-Gal expressed by the parasite was measured at 570 nm. The concentration that inhibited 50% of the parasites growth (IC_50_) was calculated through the inhibition curve of the obtained optical density values, and compared to the untreated controls. Nifurtimox was used as a positive control (IC_50_ 0.15–13.4 µM) [34,35].

#### 3.6.4. Cytotoxicity Bioassay

Vero cells were seeded in 96-well plates in RPMI 1640 medium supplemented with 10% FBS and 1% penicillin/streptomycin. The cells were allowed to grow for 24 hours before adding the test compounds, dissolved in DMSO, to final concentrations of 10, 4, 0.2 and 0.08 µg/mL. A negative control (with same volume of DMSO - added to the samples) was placed in all plates. Samples were incubated for five days before staining and examining for reduction of 3-(4,5-dimethylthiazol-2-yl)-2,5-diphenyltetrazolium bromide (MTT) and analyzed 4 hours later in a color plate reader at 570 nm [36].

## 4. Conclusions

Five compounds were isolated from the marine bacterium *Bacillus pumilus* including three indole alkaloids: 3-hydroxyacetylindole (**2**), *N*-acetyl-β-oxotryptamine (**3**) and 3-formylindole (**5**) that showed activity against *T. cruzi*. Compounds **2**, **3** and **5** showed no activity against *P. falciparum* and *L. donovani*, indicating their selectivity against *T. cruzi* parasites. Even thought alkaloids isolated in this work have been also reported from other sources different than marine bacteria; to our knowledge they constitute the first example of antitrypanosomal secondary metabolites isolated from marine heterotrophic bacteria. Given that there have not been new alternatives for the treatment of Chagas disease in half a century, the search for new antitrypanosomal compounds is of outmost importance to global programs against neglected diseases.
